# Use of a Superficial Abdominal Wall Vein in a Gravida Patient for Emergency Vascular Access

**DOI:** 10.1155/2023/1514940

**Published:** 2023-05-31

**Authors:** Christine Ha, Rotem Naftalovich, Faraz Chaudhry, Jean Eloy, Erica Spano, George Tewfik

**Affiliations:** ^1^Rutgers, The State University of New Jersey, New Jersey Medical School, Newark, NJ 07103, USA; ^2^US Army Medical Corps, Fort Sam Houston, San Antonio, TX, USA

## Abstract

Obtaining vascular access through a superficial vein of the abdominal wall of a gravida patient is an option in an emergency Cesarean surgery when other means fail. Such superficial veins may be mistaken for striae gravidarum on physical exam. A small intravenous (IV) cannula is not ideal but could save valuable time and avoid delaying induction of general anesthesia. Once the airway is secured, a larger bore IV can then be inserted while surgical exposure is undergoing. Analysis of the risks and benefits of inducing general anesthesia with a small gauge IV for a gravida patient should take into consideration risk factors for massive peripartum hemorrhage such as placental disorders (accreta, increta, precreta, abruption, or previa), presence of uterine fibroids, preeclampsia, hemolysis, elevated liver enzymes, low platelet count (HELP syndrome), severe polyhydramnios, history of grand multiparty, and bleeding disorders such as Von Willibrands and hemophilia.

## 1. Case Presentation

We report a case of vascular access through a superficial vein of the abdominal wall of a gravida patient for an emergency Cesarean surgery.

A 36-year-old multigravida intravenous (IV) drug user presented at 38 weeks gestation with rupture of membranes and contractions and in acute distress. The patient had received no prenatal care and previously underwent two cesarean deliveries. No other history was known about the patient. On examination, she was fully dilated; presentation of the fetus was complete breech at +2 to +3 station. Due to a nonreassuring fetal heart tracing, the patient was emergently transferred to the operating room (OR) for a repeat transverse cesarean delivery under general anesthesia.

In the OR, multiple attempts to obtain peripheral venous access via upper and lower extremities failed; no veins were visible and marked track marks were present on all limbs. Central line was not attempted initially because the patient was combative, anxious, and uncooperative; when external jugular attempts were made, they resulted in superficial hematomas. Insertion of a peripherally inserted central catheter (PICC) line was deemed too time-consuming, and an intraosseous kit was not readily available.

A subcutaneous vein in the abdominal wall was successful for insertion of a 24-gauge intravenous cannula near the umbilicus and general anesthesia was induced. On insertion of this IV, it was not clear whether the visualized skin darkening was indeed a superficial vein or rather from striae gravidarum. After induction, no noticeable leakage of IV fluid or propofol was noticed from the incision site. After incision, a central venous catheter was then placed in the right internal jugular vein under ultrasound guidance. The remainder of the cesarean delivery was unremarkable.

## 2. Discussion

The option of central line placement prior to induction of anesthesia was not favored because the patient was restless and the anatomy was already complicated by the superficial hematomas. If an intraosseous kit was available on the unit, it could have been an acceptable alternative for initial access, but this is a painful method which is not ideal for an awake patient. Given the circumstances of this time-sensitive situation, the option of obtaining peripheral access via the unconventional approach of an abdominal vein seemed reasonable.

A 24-gauge IV is the smallest cannula that is readily available in clinical settings. We wish to stress that such a small IV is by no means ideal for a Cesarean delivery. However, any clinical decision centers on an assessment of the risks and benefits and the clinician's judgment based on the situation. In this particular case, our preoperative assessment was that the patient lacked most of the common risk factors for massive hemorrhage during the surgery; there were no placental disorders such as accreta, increta, precreta, abruption, and previa, no uterine fibroids, no preeclampsia or hemolysis, elevated liver enzymes, low platelet count (HELP syndrome), she did not have severe polyhydramnios, history of grand multiparty, or bleeding disorders that we knew of such as Von Willibrands, hemophilia, and thrombocytopenia. She was not obese and did not have kidney disease, so we had confidence in our ability to obtain a central line within minutes after induction. Given the situation, delaying incision to obtain a central line could very well result in harm to the baby.

Subcutaneous abdominal access was reported in a cirrhotic patient with portal hypertension who required parenteral antibiotherapy. The line allowed for delivery of medications and no complications were observed [1]. Using a superficial abdomen wall veins for induction of anesthesia has been proposed as an option in children [2].

On the other hand, a similar case to the present case was reported in which a pregnant patient who was a prior IV drug user presented with serious vaginal bleeding and required an emergent Cesarean delivery. The anesthesiology team was unable to obtain venous access and ultimately opted for an inhalational induction with isoflurane without IV access for induction [3]. Other cases reported using a sevoflurane gas induction in patients with no IV access or those with needle phobia [4, 5].

Inducing anesthesia in this patient without an established venous access, would have higher risks of aspiration and airway obstruction from either laryngospasm or bronchospasm which could be triggered by the stimulation of laryngoscopy. Inducing without an IV could place the mother's life in jeopardy at the expense of her baby due to the danger of aspiration. Lack of IV access on induction would eliminate the option of a rapid sequence induction and other means for reducing aspiration risk. The ability to treat bronchospasm or laryngospasm or other complications would be limited without an IV.

Inhalation induction is more risky in obstetric patients due to the increased risk of aspiration secondary to physiological changes such as increased intragastric pressures, progesterone induced relaxation of the lower esophageal sphincter, and higher likelihood of nausea and vomiting. Furthermore, these patients' airways are more concerning since the oropharynx is more edematous and friable, complicating visualization of the vocal cords during laryngoscopy and making mask ventilation more susceptible to compromise by obstruction. The decreased functional residual capacity and increased metabolic oxygen demand can lead to rapid desaturations, and intubating without paralysis is more difficult and more likely to fail. Theoretically, if the mother consents to place her life at an increased risk for the benefit of her baby, then proceeding without an IV is an acceptable option; however, the patient needs to be well informed for an informed and proper consent. In a true such emergency scenario it may not be feasible to have a thorough discussion. The ability to inform a patient who is in distress, during a situation where seconds count, is limited and is an important factor in navigating this anesthetic plan. 

## 3. Conclusion

A superficial vein of the abdomen can, at times, be an option for venous access when conventional options fail [1]. Obtaining access in superficial veins of the abdomen can be considered even in patients undergoing Cesarean deliveries.
